# Preparation of Cylinder-Shaped Porous Sponges of Poly(L-lactic acid), Poly(DL-lactic-co-glycolic acid), and Poly(**ε**-caprolactone)

**DOI:** 10.1155/2014/106082

**Published:** 2014-02-27

**Authors:** Xiaoming He, Naoki Kawazoe, Guoping Chen

**Affiliations:** Tissue Regeneration Materials Unit, International Center for Materials Nanoarchitectonics, National Institute for Materials Science, 1-1 Namiki, Tsukuba, Ibaraki 305-0044, Japan

## Abstract

Design of mechanical skeletons of biodegradable synthetic polymers such as poly(L-lactic acid) (PLLA), poly(DL-lactic-co-glycolic acid) (PLGA), and poly(**ε**-caprolactone) (PCL) is important in the construction of the hybrid scaffolds of biodegradable synthetic polymers and naturally derived polymers such as collagen. In this study, cylinder-shaped PLLA, PLGA, and PCL sponges were prepared by the porogen leaching method using a cylinder model. The effects of polymer type, polymer fraction, cylinder height, pore size, and porosity on the mechanical properties of the cylinder-shape sponges were investigated. SEM observation showed that these cylinder-shaped sponges had evenly distributed bulk pore structures and the wall surfaces were less porous with a smaller pore size than the wall bulk pore structures. The porosity and pore size of the sponges could be controlled by the ratio and size of the porogen materials. The PLGA sponges showed superior mechanical properties than those of the PLLA and PCL sponges. Higher porosity resulted in an inferior mechanical strength. The pore size and sponge height also affected the mechanical properties. The results indicate that cylinder-shaped sponges can be tethered by choosing the appropriate polymers, size and ratio of porogen materials and dimension of sponges based on the purpose of the application.

## 1. Introduction

Porous scaffolds have been used for three-dimensional cell cultures to construct functional tissues and organs for transplantation [1–4]. The porous scaffolds provide a temporary microenvironment for the seeded cells to control cell functions, provide sufficient space for new tissue formation, and protect cells from suppression by surrounding tissues. Various porous scaffolds have been developed from both synthetic and naturally derived polymers for tissue engineering and regeneration [5–12].

In addition to the ability to allow cell adhesion, promote cell proliferation and differentiation, assemble the cells and extracellular matrices, and guide the formation of functional tissues and organs, porous scaffolds should have high mechanical strength and high porosity [13, 14]. However, porosity and mechanical strength are contradictory properties of porous scaffolds. Generally, high porosity results in low mechanical strength and vice versa. To solve the problem, biodegradable synthetic polymers such as poly(L-lactic acid) (PLLA), poly(glycolic acid) (PGA), poly(DL-lactic-co-glycolic acid) (PLGA), and poly(*ε*-caprolactone) (PCL) have been hybridized with naturally derived polymers such as collagen [15–19]. The mechanically strong synthetic polymers serve as a mechanical skeleton to support the hybrid porous scaffolds, whereas collagen sponges provide high porosity and a favorable microenvironment for cell proliferation and new tissue formation. We have used the hybridization method to prepare a new type of hybrid porous scaffolds by introducing collagen sponges into a cylinder-shaped PLLA sponge skeleton [20]. The cylinder-shaped PLLA-collagen hybrid sponges showed high mechanical strength and high porosity. Compared to block-type hybrid sponges, cylinder-shaped PLLA-collagen hybrid sponges had a higher porosity [10, 20]. The key technique in the construction of such cylinder-shaped hybrid sponges is to assemble the cylinder-shaped porous skeletons from mechanically strong synthetic polymers. Therefore, in this study, we compared three types of biodegradable polymers, PLGA, PLLA, and PCL, for the creation of a mechanically strong and stable cylinder-shaped porous skeleton. The effects of synthetic polymer type, cylinder size, and preparation conditions on the mechanical strength of the cylinder-shaped skeletons were investigated to provide important information for polymer selection and the construction of optimal porous scaffolds for tissue engineering.

## 2. Experimental and Methods

**Figure 1:**
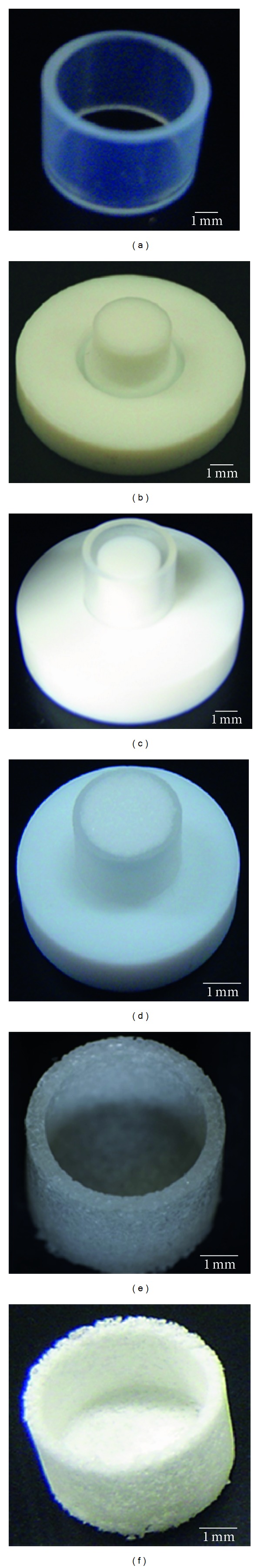
Photographs of the PFA tube (a), Teflon base (b), assembled Teflon mold (c), mixture of NaCl and PLLA filled within Teflon model (d), PLLA/NaCl cylinder (e), and cylinder-shaped PLLA sponge (f).

Cylinder-shaped sponges were prepared by the method of porogen leaching using a Teflon mold. The Teflon mold consisted of a perfluoroalkoxy (PFA) tube having an inner diameter of 12 mm and a height of 9 mm (Figure 1(a)) and a Teflon base having a pillar with a diameter of 10 mm and a height of 6 mm (Figure 1(b)). The PFA tube was plugged into the groove to construct the Teflon mold (Figure 1(c)). By using the Teflon mold, cylinder-shaped sponges having an inner diameter of 10 mm, an outer diameter of 12 mm, and a height of 7 mm can be prepared. Poly(L-lactic acid) (PLLA, weight-average molecular weight: 116, 000 ± 4700, Sigma-Aldrich, Inc., St. Louis, MO) was used to prepare the cylinder-shaped PLLA sponges. The Tg of PLLA is 47°C [21]. PLLA (1 g) was dissolved in chloroform (5 mL) in a glass tube, to which sieved sodium chloride (NaCl) particulates (9 g) were added and mixed well. Sieved NaCl particulates ranging between 90–150, 150–250, and 250–355 *μ*m were used to prepare the cylinder-shaped PLLA sponges with different pore sizes. The space between the PFA tube and the Teflon base of the Teflon mold was filled with the polymer solution/NaCl mixture by pressing the mixture into the space. The pressed mixture above the PFA tube was removed by trimming the top surface (Figure 1(d)). The filled Teflon mold was dried in an air draft for 1 day and vacuum-dried for another 3 days to allow the chloroform to evaporate completely. After drying, the Teflon mold was disassembled to remove the cylinder of the polymer/NaCl mixture from the Teflon mold (Figure 1(e)). The polymer/NaCl cylinder was immersed in deionized water to leach out the NaCl particulates. The deionized water was changed every hour. The washing was continued until the complete removal of NaCl. The cylinder-shaped PLLA sponges were dried in air after washing (Figure 1(f)).

The weight ratio of PLLA to NaCl particulates was adjusted at 1 : 4, 1 : 5, 1 : 6, 1 : 7, 1 : 8, and 1 : 9 to prepare cylinder-shaped PLLA sponges of different porosity. Either 4, 5, 6, 7, 8, or 9 grams of the sieved sodium chloride (NaCl) particulates, with a diameter ranging from 150 to 255 *μ*m, was added to the PLLA solution in chloroform (1 g/5 mL) and mixed well. The following steps were the same as those described above.

Cylinder-shaped PLLA sponges of different heights were prepared by cutting the cylinder of the polymer/NaCl mixture to a specific height during the above described preparation using 150–255 *μ*m NaCl particulates and a polymer/NaCl ratio of 1 : 9. Five heights (2, 3, 4, 5, 6, and 7 mm) of cylinder-shaped PLLA sponges were prepared to compare the effect of height on the mechanical properties.

Cylinder-shaped PLGA and PCL sponges were prepared by the same method as the cylinder-shaped PLLA sponges described above. Poly(DL-lactic-co-glycolic acid) with a copolymer ratio of 75/25 (D,L-lactic acid/glycolic acid) (PLGA, weight-average molecular weight: 109, 520 ± 1, 670, Sigma-Aldrich, Inc., St. Louis, MO) and poly(*ε*-caprolactone) (PCL, weight-average molecular weight: 261, 000 ± 2800, Sigma-Aldrich, Inc., St. Louis, MO) were used to prepare the respective cylinder-shaped PLGA and PCL sponges. The Tg of PLGA and PCL is 41 and −56°C, respectively [21]. Sieved NaCl particulates with diameter ranges of 90–150, 150–250, and 250–355 *μ*m were used. The ratio of polymer to NaCl particulates was 1 : 9. The sponge height was 7 mm.

Cross-sections of the cylinder-shaped PLLA, PLGA, and PCL sponges were made by sectioning the sponges with a razor blade. The cross-section samples were coated with platinum using a sputter coater (Sanyu Denshi, Tokyo, Japan). The wall surfaces and cross-sections of the sponges were observed by scanning electron microscopy (SEM) (JSM-6400Fs; JEOL, Tokyo, Japan).

A mercury porosimeter (Autopore IV, Shimadzu, Kyoto, Japan) was used to measure the porosity of the cylinder-shaped sponges. The sponges were cut into small pieces for the measurement.

A mechanical testing machine (TA.XTplus, Stable Micro System, UK) was used to measure the mechanical properties of the cylinder-shaped PLLA, PLGA, and PCL sponges. The dimension of each sample was measured. The dry sponges were compressed at a speed of 0.1 mm/min at room temperature. The load-deformation curves were recorded and used to calculate the elastic modulus. The calculation was done by using the software Texture Analyzer 32 provided by the same company. A total of six samples were used for the measurements of each sponge. Data were reported as mean ± standard deviation. One-way analysis of variance (ANOVA) was performed to reveal difference in Young's modulus among groups using the *t*-test. All statistical analyses were executed using StatFlex Ver. 4.2; *P* < 0.05 was considered statistically significant.

## 3. Results and Discussion

**Figure 2:**
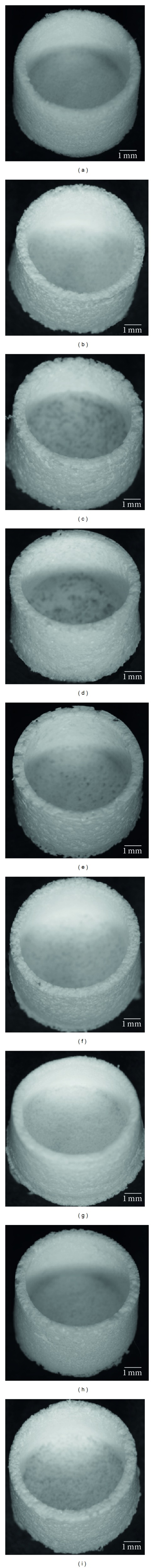
Photographs of cylinder-shaped PLLA (a–c), PLGA (d–f), and PCL (g–i) sponges prepared with NaCl particulates having a diameter range of 90–150 *μ*m (a, d, g), 150–250 *μ*m (b, e, h), and 250–355 *μ*m (c, f, i). The ratio of polymer to NaCl was 1 : 9.

Porous cylinder-shaped sponges of PLLA, PLGA, and PCL were prepared using a porogen leaching method. NaCl particulates ranging between 90–150 *μ*m, 150–250 *μ*m, and 250–355 *μ*m were used as the porogen materials and the ratio of polymer to NaCl particulates was 1 : 9.  Figure 2 shows the gross appearance of cylinder-shaped PLLA, PLGA, and PCL sponges. The height of these cylinder-like sponges was 7 mm. All of the sponges showed cylinder-like shape and were physically stable. The polymer type and the size of the NaCl particulates did not affect the gross appearance of the cylinder-shaped sponges. This method could be used to prepare cylinder-shaped sponges of different synthetic polymers by choosing the appropriate polymers.

**Figure 3:**
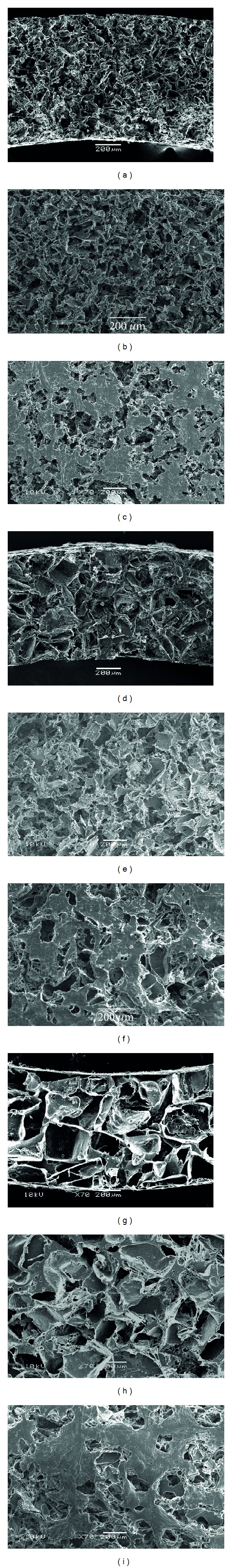
SEM microphotographs of vertical cross-sections (a, d, g), horizontal cross-sections (b, e, h), and the inner surfaces (c, f, i) of the cylinder-shaped PLLA sponges prepared with NaCl particulates having a diameter range of 90–150 *μ*m (a–c), 150–250 *μ*m (d–f), and 250–355 *μ*m (g–i). The ratio of polymer to NaCl was 1 : 9.

The porous structures of the cylinder-shaped sponges were investigated by SEM observation. The SEM images of a horizontal cross-section, a vertical cross-section, and the wall surface of cylinder-shaped PLLA sponges are shown in Figure 3. All of these cylinder-shaped sponges were highly porous with evenly distributed and interconnected pore structures in the cross-sections (bulk pore structure). The pore shapes shown in the cross-sections were similar to those of NaCl particulates. The pore size of the cylinder-shaped PLLA sponges prepared with 90–150 *μ*m, 150–250 *μ*m, and 250–355 *μ*m NaCl particulates increased with the size increase of the NaCl particulates. However, pores on the wall surfaces were fewer and smaller than those in the cross-sections. The inner wall surface and outer wall surface of the cylinder-shaped PLLA sponge showed the same pore structures.The less dense and smaller pore structure on the wall surfaces might be due to the contact effect between the NaCl particulates and the Teflon mold when the mixture of polymer solution and NaCl particulates was pressed into the Teflon mold. The angles of the cuboidal-like NaCl particulates might contact the Teflon mold and give more space for the polymer solution to fill the spaces among the angles in contact with the Teflon mold. The structures of the inner wall and the outer wall surfaces of the cylinder-shaped sponges are expected to partially protect from cell leakage during the cell seeding and cell culture. The cylinder-shaped PLGA and PCL sponges showed similar pore structures compared to that of the cylinder-shaped PLLA sponges.

**Figure 4:**
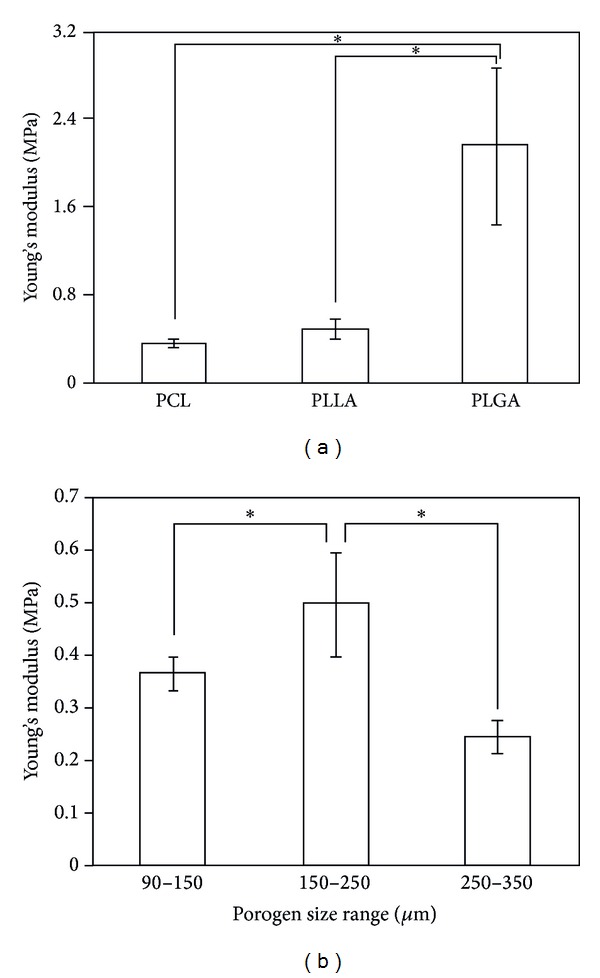
Young's modulus of cylinder-shaped PLLA, PLGA, and PCL sponges prepared with 150–250 *μ*m NaCl particulates (a) and cylinder-shaped PLLA sponges prepared with 90–150 *μ*m, 150–250 *μ*m, and 250–355 *μ*m NaCl particulates (b). The ratio of polymer to NaCl was 1 : 9. The data represent the mean ± SD of six samples.

The mechanical properties of the cylinder-shaped PLLA, PCL, and PLGA sponges were measured by a compression test. The Young's modulus of cylinder-shaped PLLA, PCL, and PLGA sponges prepared with 150–250 *μ*m NaCl particulates and a ratio of polymer to NaCl of 1 : 9 changed depending on the polymers used for the sponge preparation (Figure 4(a)). The cylinder-shaped PLGA sponge showed the highest Young's modulus, and the PCL sponge showed the lowest. PLLA is a crystalline polymer with high rigidity, while PLGA is an amorphous polymer [21]. PCL is a semicrystalline polymer and is in a rubbery state at room temperature because of its low glass transition temperature [22]. The viscoelastic property of PCL resulted in a low Young's modulus. The rigid crystalline PLLA also showed a low Young's modulus. The amorphous PLGA showed the highest Young's modulus. The low Young's modulus of cylinder-shaped PLGA sponge might be due to the difficulty in homogenous filling of PLLA/NaCl mixture in the space of Teflon mold because of the high viscosity of PLLA solution.

The size of the NaCl particulates also showed some effect on the Young's modulus. The cylinder-shaped PLLA sponges prepared with a ratio of polymer to NaCl of 1 : 9 and NaCl particles of different sizes ranging between 90–150 *μ*m, 150–250 *μ*m, and 250–355 *μ*m were compared (Figure 4(b)). The cylinder-shaped PLLA sponge prepared with 150–250 *μ*m NaCl particulates showed the highest mechanical strength, suggesting that a cylinder-like PLLA sponge with pores ranging between 150 and 250 *μ*m in size was architecturally stronger and more appropriate for applications in tissue engineering when high mechanical strength is necessary than the other tested configurations. The NaCl particles with sizes ranging between 150 and 250 *μ*m might be appropriately packed and polymer matrix could appropriately fill the spaces among the NaCl particles.

**Figure 5:**
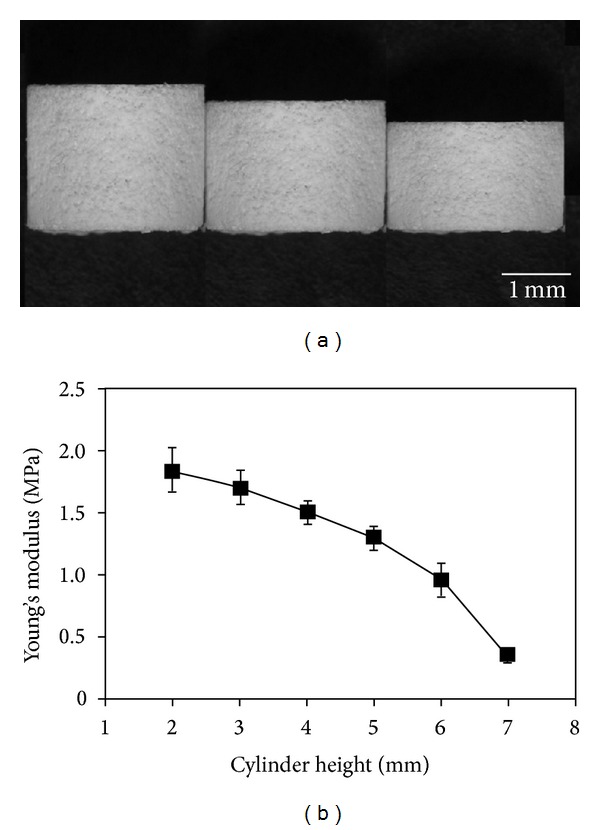
Photographs of cylinder-shaped PLLA sponges having a height of 7, 6, and 5 mm (a) and the change in Young's modulus with the sponge height (b). The data represent the mean ± SD of six samples.

The effect of the height of the cylinder-shaped PLLA sponge on the mechanical properties was investigated. Cylinder-shaped PLLA sponges with different heights of 2, 3, 4, 5, 6, and 7 mm were prepared and their mechanical strengths were compared (Figure 5). The mechanical strength decreased when the height of the cylinder-like PLLA sponge increased.

**Figure 6:**
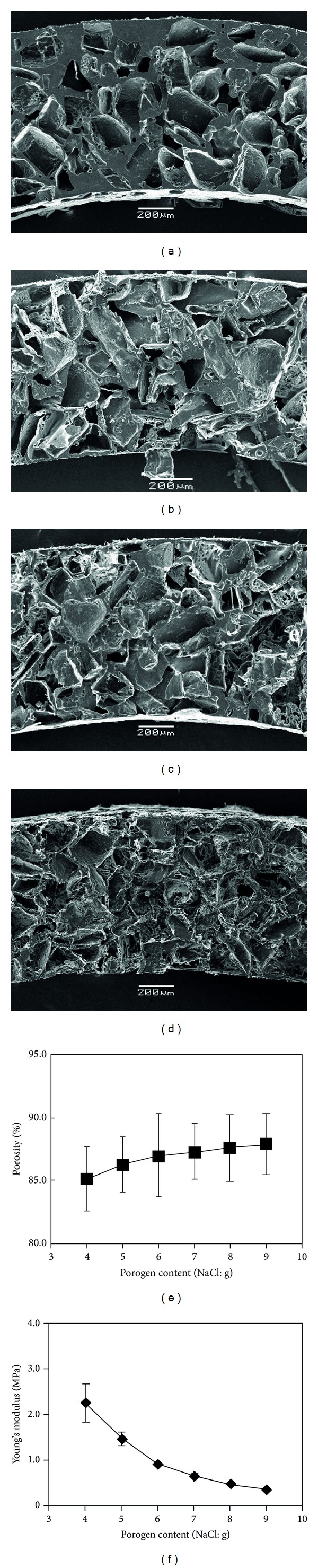
SEM microphotographs of vertical cross-sections (a–d) of the cylinder-shaped PLLA sponges prepared with 150–250 *μ*m NaCl particulates at the polymer/NaCl ratio of 1 : 4 (a), 1 : 5 (b), 1 : 6 (c) and 1 : 9 (d) and the change of porosity (e) and Young's modulus (f) with the polymer/NaCl ratio. The porosity data represent the average ± SD of three samples and the Young's modulus data represent the mean ± SD of six samples.

The mechanical property and porosity of cylinder-shaped PLLA sponges prepared with different ratio of PLLA to NaCl particulates were compared. Six types of cylinder-shaped PLLA sponges were prepared with a weight ratio of PLLA to NaCl particulates of 1 : 4, 1 : 5, 1 : 6, 1 : 7, 1 : 8, and 1 : 9. SEM microphotographs of the cross-sections of these PLLA sponges showed that more pores were observed in the cylinder-shaped PLLA sponge prepared with a higher weight fraction of NaCl particulates (Figures 6(a)–6(d)). The porosity increased when the NaCl ratio increased (Figure 6(e)). Young's modulus decreased with the increase in NaCl fraction (Figure 6(f)). A high ratio of NaCl particulates resulted in high porosity and low mechanical strength.

Mechanically strong biodegradable synthetic polymers have been hybridized with naturally derived biodegradable polymers to construct hybrid porous scaffolds [15–19]. The hybrid porous scaffolds combine the advantages of both polymers and have been used for the tissue engineering of various tissues, such as skin [23], cartilage [24, 25], bone [26], ligament [27], bladder [19], and osteochondral tissue [28]. Use of a cylinder-shaped skeleton can improve the mechanical property and simultaneously increase the porosity to provide more space for cell accommodation [20, 29]. The preparation of such cylinder-shaped skeleton is important for the formation of the hybrid structure. In this study, the effect of polymer type, pore size, and porosity on the property of the cylinder-shaped skeleton was discussed. PLLA, PLGA, and PCL can all be used to construct the cylinder-shaped sponges, while their mechanical property was dependent on the polymer type. Polymers should be selected based on the requirements of the tissue engineering application. The pore size, porosity, and cylinder height also affected the mechanical properties. The mechanical properties can be tethered by choosing the appropriate pore size and porosity and designing the dimension of the cylinder-shaped skeletons.

## 4. Conclusions

Three biodegradable synthetic polymers, PLLA, PLGA, and PCL, were used to prepare cylinder-shaped sponges using a porogen leaching method and their properties were compared. The cylinder-shaped sponges showed a porous and interconnected structure in their bulk parts, while they were less porous with smaller pores on their surfaces. The pore size and porosity could be controlled by the size and ratio of the porogen materials. The pore size, porosity, and sponge height showed some effect on the mechanical property of the sponges. The mechanical property of the cylinder-shaped sponges was also dependent on the polymer type. The PLGA sponge showed the highest mechanical strength. Therefore, the pore structure and mechanical property of the cylinder-shaped sponges could be controlled by choosing the appropriate polymers and designing the preparation conditions according to the specific application in tissue engineering.
